# Recurrent Cervical Pyogenic Spondylodiscitis Caused by Streptococcus gallolyticus subsp. pasteurianus: A Case Report

**DOI:** 10.1155/crdi/2798487

**Published:** 2026-06-12

**Authors:** Takeshi Yamashita, Katsuyuki Yoshida, Michiko Matsuzawa Adachi, Takumi Otsuki, Takahiko Fukuchi

**Affiliations:** ^1^ Department of Comprehensive Medicine 1, Division of General Medicine, Saitama Medical Center, Jichi Medical University, Saitama, Japan, jichi.ac.jp; ^2^ Department of Comprehensive Medicine 1, Division of Cardiology, Saitama Medical Center, Jichi Medical University, Saitama, Japan, jichi.ac.jp

**Keywords:** case report, cervical spondylodiscitis, pyogenic spondylodiscitis, recurrence, *Streptococcus gallolyticus* subsp. *pasteurianus*

## Abstract

Pyogenic spondylodiscitis is a rare infection, with cervical spine involvement occurring in only 3%–10% of the cases. *Streptococcus gallolyticus* subsp. *pasteurianus*, a normal gut flora component, is a rare causative agent of spinal infections. Here, we report the case of an 88‐year‐old man with cervical pyogenic spondylodiscitis caused by this organism, complicated by a clinical relapse following a standard antibiotic course. The patient presented with fever and neck pain. Blood cultures revealed *Streptococcus gallolyticus* subsp. *pasteurianus*, and cervical magnetic resonance imaging (MRI) revealed spondylodiscitis at the C4–C6 levels. The patient was treated with a 90‐day course of antibiotics (46 days intravenous and 44 days oral), leading to initial clinical and biochemical resolution. However, clinical relapse occurred on day 174, characterized by recurrent neck pain and elevated C‐reactive protein levels despite no clear signs of active inflammation on repeat MRI. Extended oral antibiotic therapy was resumed and continued for 9 months, resulting in sustained remission without surgical intervention. To our knowledge, only isolated case reports of spinal infections caused by this subspecies have been published, and no prior case has described clinical recurrence. This case suggests that standard antibiotic durations may be insufficient in selected complex or relapsing spinal infections caused by *Streptococcus gallolyticus* subsp. *pasteurianus* and highlights the importance of individualized treatment duration and long‐term surveillance.

## 1. Introduction

Pyogenic spondylodiscitis is a rare disease caused by bacterial infection of the vertebral body and intervertebral disc, with a reported annual incidence of 0.4–2.5 cases per 100,000 population [1]. Diagnosis and management of atypical spinal infections, including those caused by uncommon pathogens, present significant clinical challenges [2]. The cervical spine is a rare site of involvement, accounting for only 3%–10% of pyogenic spondylodiscitis cases, and considerably rarer than lumbar or thoracic infections [3]. Because of the narrow spinal canal and its proximity to the spinal cord and medulla oblongata, cervical infections frequently cause early neurological complications, often necessitating surgery and prolonged antibiotic therapy [3].

*Streptococcus gallolyticus* is a group D *Streptococcus* formerly known as *Streptococcus bovis*, which has been subdivided into several subspecies based on recent molecular analyses. Group D *Streptococcus* is part of the normal human gut flora, with a carrier rate of 2.5%–15% in healthy individuals [4]. Among them, *Streptococcus gallolyticus* subsp. *gallolyticus* (formerly biotype I) is strongly associated with colorectal cancer, with infective endocarditis or bacteremia occurring in 60%–85% of the patients with colorectal tumors. Contrastingly, *Streptococcus gallolyticus* subsp. *pasteurianus* (formerly biotype II/2) causes bacteremia and meningitis in neonates and infants [5, 6] and is associated with biliary tract diseases in adults; however, its association with colorectal malignancy is weak [6].

The most common causative organism of pyogenic spondylodiscitis is *Staphylococcus aureus*, followed by *Escherichia coli*, *Pseudomonas aeruginosa*, and other *Streptococci*. Spinal infections caused by *Streptococcus gallolyticus* are extremely rare. To our knowledge, the literature contains only isolated case reports of spinal infection caused by *Streptococcus gallolyticus* subsp. *pasteurianus*, including spondylodiscitis and spinal epidural abscess [7–9]. For spinal infections caused by *Streptococcus gallolyticus*, the optimal duration of therapy and potential for relapse remain poorly characterized in rare subspecies. This report aims to address this gap by presenting a detailed case in which sustained remission was achieved after prolonged multicourse antibiotic therapy.

Here, we report a case of pyogenic cervical spondylodiscitis caused by *Streptococcus gallolyticus* subsp. *pasteurianus* in an 88‐year‐old man. Considering the rarity of isolated cervical spine infections and scarcity of literature on spinal infections caused by this subspecies, we aimed to provide detailed insights into the diagnosis, treatment, and prognosis to contribute to the management of similar cases.

## 2. Case Presentation

An 88‐year‐old man presented to our emergency department with worsening neck pain. The day before admission, the patient developed fever (38.5°C), followed by the onset of right‐sided neck pain. His medical history included dilated cardiomyopathy, laparoscopic sigmoidectomy for sigmoid colon cancer, radiation monotherapy for esophageal cancer, and left nephrectomy for renal cell carcinoma. He was receiving medications for atrial fibrillation and benign prostatic hyperplasia.

Upon admission, after taking acetaminophen, his vital signs were as follows: temperature, 37.2°C; blood pressure, 119/98 mmHg; heart rate, 94 beats/min; respiratory rate, 18 breaths/min; and oxygen saturation, 100% on room air. Physical examination revealed neck tenderness on flexion but not rotation. The patient’s laboratory results upon admission are summarized in Table 1. These findings were significant for an inflammatory response, with a white blood cell count of 12,410/μL and C‐reactive protein (CRP) of 14.08 mg/dL, and evidence of acute‐on‐chronic kidney injury (urea nitrogen: 31 mg/dL and creatinine: 1.75 mg/dL). Chest, abdominal, and pelvic noncontrast computed tomography (CT) showed no clear cause of fever. After blood cultures were drawn, the patient was admitted for observation and treated symptomatically with acetaminophen.

**TABLE 1:** Laboratory data upon admission.

Parameter	Admission	Reference range
*Complete blood count*		
White blood cell	12.41	3.5–9.1 × 10^3^/μL
Neutrophils	84.9	40%–74%
Lymphocytes	8.3	19%–48%
Monocytes	5.2	3.4%–9%
Eosinophils	1.4	0%–7%
Red blood cell	476	376–500 × 10^4^/μL
Hemoglobin	14.0	11.3–15.2 g/dL
Hematocrit	41.8	33.4%–44.9%
Mean corpuscular volume	87.8	79–100 fL
Platelet	11.9	13–36.9 × 10^4^/μL

*Coagulation tests*		
PT‐INR	1.64	0.9–1.2
APTT	33.8	28.5–40.9 s
D‐dimer	1.4	0–1 μg/mL

*Urinalysis and sediments*		
Gravity	1.020	1.005–1.025
pH	5.5	5–7.5
Protein	—	Negative
Glucose	—	Negative
Occult blood	+	Negative
Nitrite	—	Negative
White blood cell	+	Negative

*Biochemistry*		
Total protein	6.5	6.6–8.1 g/dL
Albumin	3.8	4.1–5.1 g/dL
Total bilirubin	1.37	0.4–1.5 mg/dL
Direct bilirubin	0.53	0.05–0.23 mg/dL
AST	35	13–30 U/L
ALT	19	7–23 U/L
LD	226	124–222 U/L
Creatine kinase	589	41–153 U/L
ALP	114	106–322 U/L
γ‐GT	15	9–32 U/L
C‐reactive protein	14.08	0.00–0.14 mg/dL
Urea nitrogen	31	8–20 mg/dL
Creatinine	1.75	0.46–0.79 mg/dL
Sodium	136	138–145 mmol/L
Potassium	4.5	3.6–4.8 mmol/L
Chloride	99	100–110 mmol/L
Corrected calcium	9.2	8.4–10.1 mg/dL
Inorganic phosphorus	3.1	2.7–4.6 mg/dL
Magnesium	2.0	1.7–2.5 mg/dL
Plasma glucose	132	70–109 mg/dL
Brain natriuretic peptide	165	0–18.4 pg/mL

*Note:* AST, aspartate aminotransferase; ALT, alanine aminotransferase; ALP, alkaline phosphatase; PT‐INR, international normalized ratio of prothrombin time.

Abbreviations: APTT, activated partial thromboplastin time; GGT, gamma‐glutamyl transferase; HPF, high‐power field; LD, lactate dehydrogenase; n/a, not available.

On hospital day 2, lumbar puncture showed no significant pleocytosis and blood cultures grew *Streptococcus*‐like organisms, and treatment with intravenous ceftriaxone (2 g every 24 h) was initiated. On hospital day 4, both blood culture sets were identified as *Streptococcus gallolyticus* subsp. *pasteurianus* and the antibiotic was switched to intravenous ampicillin (2 g every 6 h). Antimicrobial susceptibility testing revealed the isolate to be susceptible to penicillin, with a minimum inhibitory concentration (MIC) of ≤ 0.063 μg/mL, and ampicillin (MIC ≤ 0.125 μg/mL) and ceftriaxone (MIC ≤ 0.5 μg/mL) (Table 2). Transthoracic echocardiography showed diffuse hypokinesis but no obvious vegetation. Repeat blood cultures were confirmed to be negative. On hospital day 6, colonoscopy revealed several small colonic polyps (4–6 mm). Esophagogastroduodenoscopy performed 6 months previously revealed atrophic gastritis and gastric polyps.

**TABLE 2:** Antimicrobial susceptibility testing.

**Blood culture**		
***Streptococcus gallolyticus* subsp. *pasteurianus***		
**Antimicrobial susceptibility**		
**Antimicrobial**	**Interpretation**	**MIC (μg/mL)**

Penicillin G	Susceptible	≤ 0.063
Ampicillin	Susceptible	≤ 0.125
Cefotaxime	Susceptible	≤ 0.125
Ceftriaxone	Susceptible	≤ 0.5
Meropenem	Susceptible	≤ 0.125
Erythromycin	Susceptible	≤ 0.125
Clindamycin	Susceptible	≤ 0.25
Azithromycin	Susceptible	0.5
Levofloxacin	Susceptible	1
Vancomycin	Susceptible	0.25

Abbreviation: MIC, minimum inhibitory concentration.

On hospital day 10, cervical spine magnetic resonance imaging (MRI) was suggestive of pyogenic spondylodiscitis at the C4–C6 levels, showing high signal intensity in the vertebral bodies and intervertebral discs on T2‐weighted and short‐tau inversion recovery (STIR) images with corresponding low signal intensity on T1‐weighted images (Figure 1). Consequently, pyogenic spondylodiscitis due to *Streptococcus gallolyticus* subsp. *pasteurianus* was diagnosed. The patient completed a 46‐day course of intravenous antibiotics and was discharged. After discharge, the patient was treated with oral ampicillin (2 g/day in divided doses) and probenecid (1 g/day in divided doses). On day 90, antibiotic therapy was discontinued after his CRP level and erythrocyte sedimentation rate (ESR) normalized.

**FIGURE 1:**
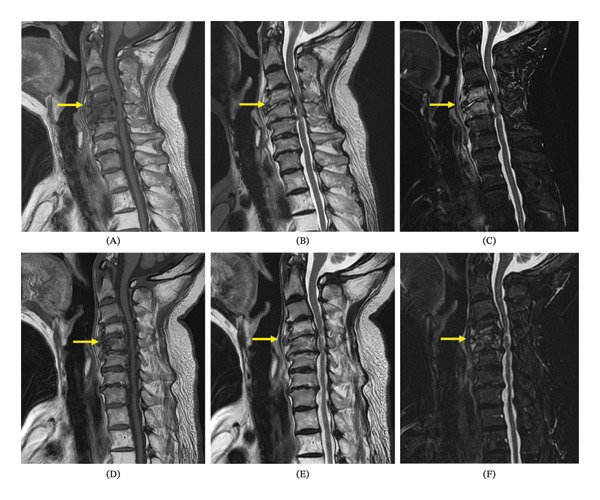
Cervical spine magnetic resonance imaging upon admission and at clinical relapse. (A–C) Upon admission (day 10). Sagittal T1‐weighted (A), T2‐weighted (B), and short‐tau inversion recovery (STIR) (C) images show abnormal signal intensity at the C4‐C6 levels. High‐signal intensity is noted in the C4–C6 vertebral bodies, C4/5 and C5/6 intervertebral discs, and posterior longitudinal ligament on the T2‐weighted and STIR images (arrows), with corresponding low‐signal intensity on the T1‐weighted image (arrows). (D–F) At clinical relapse (day 174). Corresponding sagittal T1‐weighted (D), T2‐weighted (E), and STIR (F) images. Despite the patient’s recurrent neck pain and elevated C‐reactive protein, the previously noted high‐signal abnormalities on the STIR image (F) have been resolved, and there are no clear signs of active inflammation.

The patient initially remained asymptomatic; however, 174 days after admission, he presented with recurrent neck pain. Blood tests revealed elevated CRP of 1.69 mg/dL. Although a repeat cervical MRI with STIR sequence did not show any clear high signal intensity, clinical relapse was suspected based on recurrent focal neck pain and elevated CRP levels. The neck pain did not improve despite treatment with acetaminophen/tramadol before restarting antibiotics. On day 188, oral ampicillin and probenecid were restarted, after which the neck pain gradually improved. Therefore, the recurrent symptoms and inflammatory response were considered more consistent with relapse of spondylodiscitis than with noninfectious musculoskeletal pain. Oral antibiotic treatment was continued for nine months and stopped on day 466 after normalization of CRP levels and ESR was confirmed. The patient’s clinical course is summarized in Figure 2. During follow‐up, no neurological deficits or long‐term functional decline were observed. Repeat MRI was performed only at the time of suspected relapse, and subsequent follow‐up was mainly based on clinical symptoms and inflammatory markers, including CRP and ESR. At 9 months after completion of antibiotic therapy, the patient remained free from recurrence.

**FIGURE 2:**
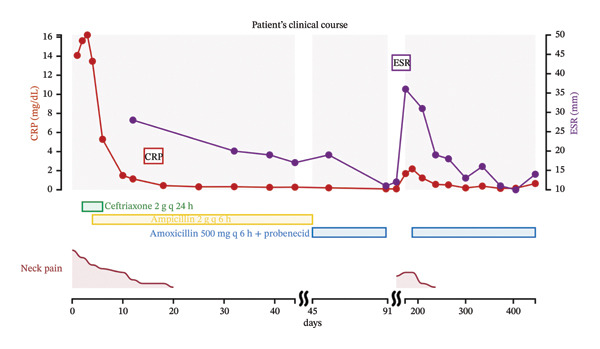
Patient’s clinical course. Trends in C‐reactive protein and erythrocyte sedimentation rate, along with the timeline of antibiotic administration and patient’s subjective neck pain.

## 3. Discussion

This case illustrates a rare cervical spinal infection caused by *Streptococcus gallolyticus* subsp. *pasteurianus*. A review of the literature identified only isolated case reports, and there is no previously documented clinical recurrence [7–9] (Table 3). Because cervical involvement accounts for only 3%–10% of pyogenic spinal infections [3], such cases require cautious management. The anatomical constraints of the cervical region, including the narrow spinal canal and proximity to critical neurological structures, necessitate aggressive management approaches combining surgical intervention with prolonged antibiotic therapy [3, 10]. Our patient achieved remission through conservative management alone, suggesting that carefully monitored prolonged therapy may be effective in selected patients despite anatomically high‐risk infections.

**TABLE 3:** Reported cases of spinal infection caused by *Streptococcus gallolyticus* subsp. *pasteurianus*.

Case (author, year)	Age/sex	Infection site	Diagnosis	Complications	Treatment	Relapse	Outcome
Mukherjee et al. (2022)	70s/Female	Thoracolumbar (T12–L1)	Spinal epidural abscess Spondylitis	Diabetic ketoacidosis	Surgical decompression Antibiotics	No	Fatal
García‐País et al. (2016)	89/Female	Lumbar (L2‐L3)	Spondylodiscitis, paravertebral abscess	Infective endocarditis	Antibiotics	No	Fatal
Chand et al. (2016)	70/Male	Lumbosacral (L3‐L4, L5–S1) Cervical (C5‐C6)	Osteomyelitis, discitis	Infective endocarditis Colon adenoma	Antibiotics Surgery(valves, spine, colon)	No	Discharged
Present case	88/Male	Cervical (C4–C6)	Spondylodiscitis	None	9 months of antibiotics	Yes	Healing

*Note:* Summary of previously reported cases and the present case. The current case is the only reported case of relapse that was successfully treated with prolonged antibiotic therapy.

The clinical context of *Streptococcus gallolyticus* subsp. *pasteurianus* bacteremia warrants subspecies‐specific considerations. Unlike *Streptococcus gallolyticus* subsp. *gallolyticus*, which is strongly associated with colorectal neoplasms (60%–85% of the cases) [4], the *pasteurianus* subspecies demonstrates weaker associations with colorectal malignancy [6]. Previous reviews have shown that *Streptococcus gallolyticus* subsp*. gallolyticus* is most closely associated with colorectal neoplasia, whereas *Streptococcus gallolyticus* subsp. *pasteurianus* is more often associated with hepatobiliary or urinary tract infections [11]. Recent molecular studies have shown that *Streptococcus gallolyticus* subsp. *pasteurianus* is widely distributed among livestock and frequently carries macrolide‐ and tetracycline‐resistance genes [12]. This subspecies is characteristically linked to invasive diseases in neonates and infants [13] and biliary tract infections in adults [6]. Although only small polyps were found, colonoscopy was warranted because of the risk of residual malignancy.

A notable aspect of this case was the occurrence of clinical relapse after apparent initial resolution. Recurrence on day 174, despite normalized inflammatory markers on day 90, illustrates the difficulty in accurately assessing the treatment response in spinal infections. Relapse was defined clinically by recurrent focal neck pain, elevated CRP levels, lack of improvement with analgesic therapy, and subsequent improvement after resumption of antibiotics, despite no clear signs of active inflammation on repeat MRI. Although noninfectious causes of neck pain and CRP elevation, such as degenerative or mechanical pain, were considered, the clinical course was more consistent with the relapse of spondylodiscitis. MRI findings may lag behind clinical disease activity and do not reliably exclude relapse, particularly when clinical symptoms and inflammatory markers suggest ongoing infection. This is consistent with previous reports indicating that MRI findings do not always correlate with clinical outcomes in bacterial vertebral osteomyelitis [14]. Our experience suggests the importance of integrating clinical symptoms, inflammatory biomarkers, and imaging findings when evaluating possible relapse of pyogenic spondylodiscitis [15].

Our patient received nine months of oral therapy after relapse, which exceeded the conventional 6–12‐week recommendation [16, 17]. The prolonged regimen was individualized because relapse occurred after an apparently adequate initial 90‐day antibiotic course, the infection involved the cervical spine in an elderly patient with multiple comorbidities, and CRP and ESR normalized only gradually after treatment was resumed. As oral ampicillin plus probenecid was well tolerated, therapy was continued until sustained clinical improvement and normalization of inflammatory markers were confirmed. However, the optimal treatment duration remains uncertain in complex or relapsing cases. Thus, the prolonged treatment duration in this patient was determined by the individual clinical course and treatment response rather than by a pathogen‐specific protocol.

Several factors may have contributed to treatment challenges. The patient’s advanced age (88 years) and multiple comorbidities, including previous malignancies and renal dysfunction, together with the cervical location, likely created multiple factors complicating treatment. Although direct evidence of subspecies‐specific virulence or treatment resistance in *Streptococcus gallolyticus* subsp. *pasteurianus* remains limited, genomic analyses of the *Streptococcus gallolyticus* complex have revealed adhesion and surface‐protein diversity that may favor bacterial persistence [18, 19]. Clinical studies of pyogenic spondylodiscitis have identified host and infection‐related predictors of poor outcomes (advanced age, renal impairment, and multiple vertebral involvement) [20, 21]. These observations suggest that bacterial and host factors may have contributed to the prolonged disease course in our patient although a specific virulence mechanism could not be established. This case highlights the importance of long‐term surveillance of spinal infections. The relatively late relapse on day 174 emphasizes that clinical vigilance must extend beyond the immediate treatment period. Regular monitoring of inflammatory markers and clinical symptoms for several months after treatment is prudent, particularly for infections in challenging anatomical locations or those caused by uncommon pathogens.

Furthermore, this case contributes to the limited literature on *Streptococcus gallolyticus* subsp. *pasteurianus* spinal infections, while highlighting the heterogeneity of treatment responses, even within rare pathogen categories. The successful outcome achieved through extended conservative therapy provides an important therapeutic option for clinicians managing similar cases, particularly in patients for whom surgical intervention carries a prohibitive risk.

The present case has several limitations. First, no tissue culture or vertebral biopsy was performed; therefore, the diagnosis was based on positive blood cultures from two sets, compatible cervical MRI findings, and the absence of an alternative infectious focus. The absence of tissue biopsy may have reduced the diagnostic certainty, particularly when assessing suspected relapse. Biopsy was not performed because the risk of procedure‐related complications was considered to outweigh the potential diagnostic benefit in this elderly patient with multiple comorbidities and cervical spine involvement. Second, the portal of bacterial entry could not be definitively identified. Third, the decision to extend antibiotic therapy was based on clinical judgment and biomarker trends rather than on established pathogen‐specific protocols.

Overall, this case suggests that spinal infections caused by *Streptococcus gallolyticus* subsp. *pasteurianus* may relapse even after apparently adequate therapy in selected patients. In our patient, prolonged oral ampicillin plus probenecid after relapse was associated with sustained remission, suggesting that carefully monitored, prolonged oral therapy may be a reasonable option in selected complex or relapsing cases.

## 4. Conclusion

Recurrent cervical pyogenic spondylodiscitis due to *Streptococcus gallolyticus* subsp. *pasteurianus* is extremely rare. Clinicians should consider this pathogen in patients with cervical spine infections and appropriate risk factors and perform a thorough search for predisposing conditions. This case may suggest that individualized extension of antibiotic treatment and long‐term follow‐up are warranted in selected complex or relapsing cases although optimal treatment duration remains uncertain.

## Author Contributions

Takeshi Yamashita managed the patient and drafted the manuscript. Katsuyuki Yoshida, Michiko Matsuzawa Adachi, Takumi Otsuki, and Takahiko Fukuchi contributed to clinical management and manuscript revision.

## Funding

The authors received no specific funding for this work.

## Disclosure

All authors have approved the final manuscript.

## Ethics Statement

Ethical approval was waived in accordance with the institutional policy of Jichi Medical University Saitama Medical Center because this study was a single case report. All the patients allowed personal data processing, and informed consent was obtained from all individual participants included in the study.

## Conflicts of Interest

The authors declare no conflicts of interest.

## Data Availability

Data are available from the corresponding author upon reasonable request. No further changes are required.
